# *Rhodiola crenulata* and Its Bioactive Components, Salidroside and Tyrosol, Reverse the Hypoxia-Induced Reduction of Plasma-Membrane-Associated Na,K-ATPase Expression via Inhibition of ROS-AMPK-PKC**ξ** Pathway

**DOI:** 10.1155/2013/284150

**Published:** 2013-06-06

**Authors:** Shih-Yu Lee, Li-Shian Shi, Hsin Chu, Min-Hui Li, Cheng-Wen Ho, Feng-Yi Lai, Chih-Yang Huang, Tsu-Chung Chang

**Affiliations:** ^1^Graduate Institute of Medical Sciences, National Defense Medical Center, Taipei 114, Taiwan; ^2^Institute of Aerospace and Undersea Medicine, National Defense Medical Center, Taipei 114, Taiwan; ^3^Department of Biotechnology, National Formosa University, Yunlin 632, Taiwan; ^4^Department of Biochemistry, National Defense Medical Center, P.O. Box 90048-501, Nei-hu, Taipei 114, Taiwan; ^5^Department of Health and Nutrition Biotechnology, Asia University, Taichung 413, Taiwan; ^6^Graduate Institute of Basic Medical Science, China Medical University, Taichung 400, Taiwan

## Abstract

Exposure to hypoxia leads to impaired pulmonary sodium transport, which is associated with Na,K-ATPase dysfunction in the alveolar epithelium. The present study is designed to examine the effect and mechanism of *Rhodiola crenulata* extract (RCE) and its bioactive components on hypoxia-mediated Na,K-ATPase endocytosis. A549 cells were exposed to hypoxia in the presence or absence of RCE, salidroside, or tyrosol. The generation of intracellular ROS was measured by using the fluorescent probe DCFH-DA, and the endocytosis was determined by measuring the expression level of Na,K-ATPase in the PM fraction. Rats exposed to a hypobaric hypoxia chamber were used to investigate the efficacy and underlying mechanism of RCE *in vivo*. Our results showed that RCE and its bioactive compounds significantly prevented the hypoxia-mediated endocytosis of Na,K-ATPase via the inhibition of the ROS-AMPK-PKC**ζ** pathway in A549 cells. Furthermore, RCE also showed a comparable preventive effect on the reduction of Na,K-ATPase endocytosis and inhibition of AMPK-PKC**ξ** pathway in the rodent model. Our study is the first to offer substantial evidence to support the efficacy of *Rhodiola* products against hypoxia-associated Na,K-ATPase endocytosis and clarify the ethnopharmacological relevance of *Rhodiola crenulata* as a popular folk medicine for high-altitude illness.

## 1. Introduction 

In normal physiology, there is a homeostasis between alveolar fluid secretion and clearance. Alveolar fluid clearance (AFC) mainly depends on sodium transport in the alveolar epithelium. The apical surface is cleared primarily via the epithelial sodium channel (ENaC), a predominant transepithelial sodium channel in organs including the lungs, kidneys, and colon, which is responsible for 40–60% of reabsorption. On the other hand, sodium is actively pumped out by Na,K-ATPase which is located in the basolateral plasma membrane to the pulmonary interstitium. This, in turn, leads to an osmotic gradient in the transepithelial membrane that water follows [1, 2]. These transporters are essential for the maintenance of transcellular fluid transport. The malfunction of these transporters has been associated with dysfunctions in AFC, alveolar flooding, and edema. For example, alveolar hypoxia, a common phenomenon when abruptly relocating nonacclimatized individuals to a high altitude, is thought to be involved in impaired transalveolar fluid transport. The excessive fluid that subsequently accumulates in alveoli exaggerates alveolar hypoxia and impairs gas exchange, and these processes are associated with the pathological progression of high altitude pulmonary edema (HAPE), the most lethal form of high-altitude illnesses [3, 4].

Recent reports have documented that Na,K-ATPase plays a key role in the maintenance of AFC. It was shown that both the inhibition and knockdown of Na,K-ATPase expression significantly diminished AFC in rodent models [5, 6]. In contrast, adenovirus-mediated overexpression of Na,K-ATPase in alveoli obviously improves AFC in rats [7]. It should be noted that AFC was shown to be associated only with the protein abundance of Na,K-ATPase in the plasma membrane (PM) rather than the total amount in both cell and rodent models [6–8]. Furthermore, a large body of evidence also indicated that hypoxia is associated with impaired AFC, including mortality in studies using both *in vivo* and *in vitro* models as well as clinical studies. The phenomena that manifest during hypoxia are due in part to the hypoxia-associated malfunction of active sodium transport that results from the declined activity and expression of Na,K-ATPase [9–12]. 

Exposure to hypoxia leads to mitochondrial complex III dysfunction that results in the intensive generation of intracellular ROS, which in turn activates the AMP-activated protein kinase (AMPK). Through the direct activation of the protein kinase C*ζ* (PKC*ζ*) pathway, the activated AMPK leads to the phosphorylation of Na,K-ATPase, which serves as the triggering signal for its endocytosis from the basolateral membrane, as indicated in both A549 cells and the alveolar epithelial type II cells of rats [13, 14].

**Figure 1:**
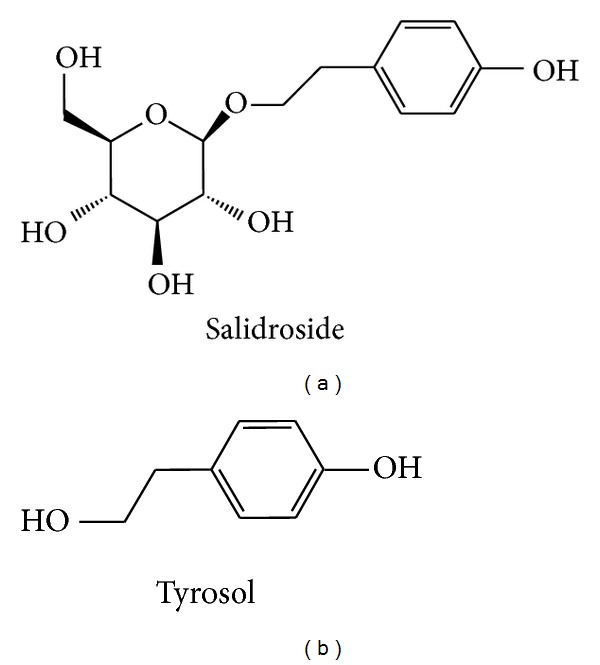
Structures of salidroside (a) and tyrosol (b).

*Rhodiola* species, a popular folk medicine in Asian and eastern European countries, have been used for the prevention of high-altitude illness and are regarded as traditional phytoadaptogens against environmental challenges in Tibet. These species show beneficial properties, including antidepression, neuroprotective, cardioprotective, antifatigue, antihypoxia, and hepatoprotective activities. In addition, *Rhodiola* species have also been reported to promote longevity and work productivity [15]. Salidroside and tyrosol (Figure 1) were shown to contribute to *Rhodiola crenulata*'s benefits, which include antioxidant, antidepression, antifatigue, and anti-inflammatory activities [16, 17]. In our previous study [18], we demonstrated that *Rhodiola crenulata* extract (RCE) displayed excellent antioxidant activity and attenuated several indicators of pulmonary edema induced by hypobaric hypoxia in a rodent model. However, the effect and mechanism of *Rhodiola crenulata* on sodium transport in alveolar cells still needs to be elucidated. The present study attempted to examine whether RCE, salidroside, and tyrosol are efficacious in preventing the hypoxia-mediated Na,K-ATPase endocytosis and to clarify the underlying mechanisms using both A549 cells and rodent models.

## 2. Materials and Methods

### 2.1. Materials

*Rhodiola crenulata* were purchased from Chuang Song Zong Pharmaceutical Co., Ltd (Taichung, Taiwan). The plant was authenticated by Dr. H. C. Lin in the National Defense Medical Center, where a voucher specimen (NDMCP no. 1000901) has been deposited. Compound C and GF10923X from TOCRIS (Bristol, UK), N-acetyl-l-cysteine (NAC) from Sigma (St. Louis, MO, USA), and tyrosol, which was a kind gift from Dr. Li-Shian Shi (National Formosa University, Yunlin, Taiwan), were also used in the study.

### 2.2. Preparation of Salidroside and *Rhodiola crenulata* Extract

The dried roots of *Rhodiola crenulata *(2.0 kg) were ground into a fine powder and soaked (24 h) with 95% ethanol (10 L × 2), then refluxed with 95% ethanol (10 L × 1). The evaporation of the solvent under reduced pressure provided 320.24 g of RCE, which was successively partitioned between *n*-hexane-90% MeOH, CHCl_3_-H_2_O, EtOAc-H_2_O, and *n*-BuOH-H_2_O to yield *n*-hexane (32.89 g), CHCl_3_ (10.08 g), EtOAc (76.15 g), *n*-BuOH (101.26 g), and H_2_O (99.86 g) fractions. The *n*-BuOH fraction (101.30 g) was applied to Diaion HP-20 column chromatography and eluted with an H_2_O-MeOH gradient system to give 11 fractions. Fraction 5 (12.34 g) was chromatographed on silica gel and eluted with CHCl_3_-MeOH (12 : 1) to give compound **1** (1.24 g). The structure of compound **1** was identified as salidroside by comparing it to the spectral data in the literature [19].

### 2.3. Cell Culture

 A549 cells (the human lung adenocarcinoma epithelial cell line), a widely used cell model for the investigation of pulmonary transepithelial sodium transport [14], were purchased from American Type Culture Collection (ATCC, Manassas, VA, USA) and maintained in Dulbecco's modified Eagle medium (DMEM) containing 100 IU/mL penicillin, 100 *μ*g/mL streptomycin, and 7% fetal calf serum (Gibco, Gaithersburg, MD, USA) in 5% CO_2_ at 37°C. The medium was changed every two to three days, and the cells were trypsinized and subcultured when the cells were grown to approximately 80% confluence. All of the experiments were carried out using cells with 3–8 passages. To allow cell differentiation for the experiments, the A549 cells were cultured for another 7 days after reaching confluence.

### 2.4. Cell Viability Assay

A cell viability assay was performed using a cell counting kit-8 (CCK-8, Dojindo, Japan), as described previously [20]. Briefly, 5 × 10^3^ cells were seeded in each well of the 96-well plates and allowed to attach overnight. Subsequently, the cells were pretreated with different concentrations of RCE, salidroside, or tyrosol for 30 min before exposure to a hypoxia chamber with a 1% O_2_, 5% CO_2_, and 94% N_2_ hypoxic environment. After 24 h of hypoxic exposure, the original medium was removed and 10 *μ*L of CCK-8 in 90 *μ*L of PBS was added to react with cells for 2 h. The absorbance was examined at 450 nm by using a spectrophotometer. 

### 2.5. Measurement of Intracellular ROS

The quantitation of intracellular ROS was measured using the fluorescent probe 2,7-dichlorofluorescein diacetate (DCFH-DA), as previously described [21]. Briefly, the cells were pretreated with different concentrations of RCE and its bioactive components in a 96-well microplate for 30 min followed by exposure to hypoxia (1% O_2_) for 24 h. The original medium was removed, and DCFH-DA in PBS (10 mM) was added to each well at 37°C for 15 min in the incubator. Subsequently, the cells were lysed with lysis buffer and centrifuged at 14,000 rpm for 1 min. The supernatant was collected and measured at an excitation and emission wavelength of 485 and 538 nm, respectively, using a fluorescence microtiter plate reader (POLARstar Galaxy; BMG Labtechnologies, Offenburg, Germany). The results were normalized by the protein concentration of each sample, which was determined by using a BCA (bicinchoninic acid) protein assay kit (Pierce, Rockford, IL, USA). 

### 2.6. PM and Post-Plasma Membrane (PPM) Isolation

 The PM fraction of A549 cells and rat lung tissues was isolated as previously described [22]. Briefly, the cells and tissues were lysed in buffer A (50 mM Tris-Cl and 0.5 mM DTT, pH 8.0) supplemented with 0.1% (v/v) Nonidet P-40 (NP-40), protease inhibitors, and phosphatase inhibitors. Subsequently, the cell lysate was passed through a 1 mL syringe fitted with a 25-gauge needle 10 times and centrifuged at 1000 ×g for 10 min. The precipitate was suspended in buffer A without NP-40 and recentrifuged at 1000 ×g for 10 min. Thereafter, the precipitate was resuspended again in buffer A with 1% NP-40 on ice for 1 h and then centrifuged at 16000 ×g for 20 min. The supernatant was collected as a PM fraction. In the meantime, the supernatants collected at the first and second steps were pooled and centrifuged at 16000 ×g for 20 min. Thereafter the supernatant was collected as the PPM fraction.

### 2.7. Hypobaric Hypoxia Exposure in Rats

The method used was described previously [23] and was approved by the Institutional Animal Care and Use Committee of the National Defense Medical Center (IACUC-11-055). The rats were exposed to a simulated altitude of 8000 m in a hypobaric hypoxia chamber at room temperature for 9 h. The rats were gavaged with RCE suspended in 1 mL saline via a gastric cannula once daily for 3 days before exposure to hypoxia. The same steps were followed for the normoxic control group (saline). After hypoxic exposure, the rats were anesthetized with an intraperitoneal injection of sodium pentobarbital (10 mg/kg), the abdomen was opened, and the lung was dissected. The samples were stored at −80°C until use. 

### 2.8. Western Blot Analysis

The expression of specific proteins in A549 cells and rat lung tissues was quantified as described previously [18]. The A549 cells and lung tissue were homogenized in RIPA buffer or buffer A with protease and phosphatase inhibitors. Equal amounts of protein from each sample were separated using sodium dodecyl sulfate (SDS)-10% polyacrylamide gel electrophoresis (PAGE) and transblotted onto polyvinylidene difluoride (PVDF) membranes (Millipore, Bedford, MA, USA). Immunoblotting was performed with antibodies for *α*1-Na,K-ATPase (Novus Biologicals, Littleton, CO, USA), p-AMPK (T172), AMPK (Cell Signaling Tech, Danvers, MA, USA), PKC*ζ* (GeneTex, San Antonio, TX, USA), superoxide dismutase 2 (SOD2), clathrin, glutathione peroxidase 2 (GPx2) (Santa Cruz, CA, USA), and *β*-actin (Chemicon, Temecula, CA, USA). The signals were visualized with an enhanced chemiluminescence kit (ECL, Amersham, UK) followed by exposure to X-ray films. 

### 2.9. Statistical Analysis

All of the data represent the mean ± S.E.M. Significant differences among group means were determined with a one-way ANOVA with repeated measures followed by Newman-Keuls test using SPSS 14.0 for Windows software. *P* < 0.05 was considered statistically significant.

## 3. Results

### 3.1. RCE, Salidroside, and Tyrosol All Suppress the Hypoxia-Induced Intracellular ROS Burden with No Significant Cytotoxicity in A549 Cells

**Figure 2:**
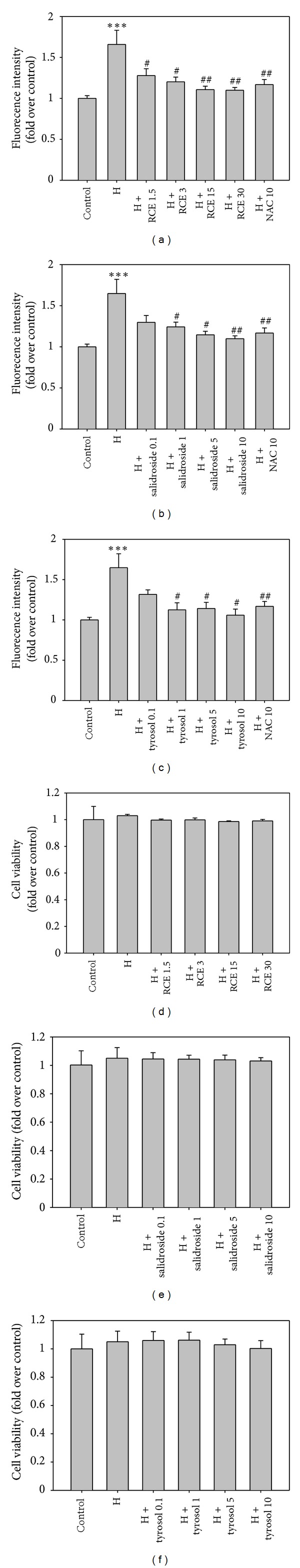
RCE, salidroside, and tyrosol significantly attenuate the hypoxia-induced generation of intracellular ROS and show no cytotoxicity in A549 cells. The cells were preincubated with the indicated concentrations of RCE, salidroside, and tyrosol for 30 min before hypoxic exposure (1% O_2_) for 24 h. The intracellular ROS was measured via the DCFH-DA (10 mM) fluorescence method ((a), (b), and (c)). The cell viability under hypoxia in the presence or absence of *Rhodiola* products was examined using a CCK-8 reagent kit ((d), (e), and (f)). The concentration units of RCE, salidroside, and tyrosol are *μ*g/mL and *μ*M, respectively. NAC (10 *μ*M) served as a positive control. The results represent the mean ± SEM of six independent experiments. *P < 0.05; **P < 0.01; ***P < 0.001 versus control; ^#^ *P* < 0.05; ^##^ *P* < 0.01; ^###^ *P* < 0.001 versus hypoxia (H).

After exposure to hypoxia for 24 h, the intracellular ROS level in A549 cells was significantly elevated compared with the control (1.65 ± 0.17-fold, *P* < 0.001) (Figures 2(a), 2(b), and 2(c)). Pretreatment with different concentrations of RCE (1.5, 3.0, 15, and 30 *μ*g/mL) decreased the hypoxia-induced production of intracellular ROS in a dose-dependent manner (1.27 ± 0.07, 1.20 ± 0.05, 1.11 ± 0.05, and 1.09 ± 0.03-fold change relative to controls; *P* < 0.05, *P* < 0.05, *P* < 0.01, and *P* < 0.01, resp.; Figure 2(a)). Consistently, both salidroside and tyrosol (0.1, 1.0, 5.0, and 10 *μ*M) exerted a similar anti-ROS efficacy in A549 cells under hypoxia in a dose-dependent manner. As shown in Figure 2(b), the results for salidroside are 1.30 ± 0.08, 1.24 ± 0.06, 1.15 ± 0.04, and 1.10 ± 0.03-fold compared to the control (NS (not significant), *P* < 0.05, *P* < 0.05, and *P* < 0.01, resp.). Similarly, the results for tyrosol are 1.32 ± 0.06, 1.13 ± 0.09, 1.14 ± 0.08, and 1.06 ± 0.08-fold compared to the control (NS, *P* < 0.05, *P* < 0.05, and *P* < 0.05, resp.) (Figure 2(c)). As a positive control, NAC (a thiol-reducing agent) also showed the ability to alleviate the hypoxia-induced production of intracellular ROS (1.17 ± 0.06-fold compared to the control, *P* < 0.01). Furthermore, RCE, salidroside, and tyrosol showed no significant cytotoxicity in the dose range studied under our experimental conditions (Figures 2(d), 2(e), and 2(f)).

### 3.2. RCE, Salidroside, and Tyrosol Prevent Hypoxia-Mediated Na,K-ATPase Endocytosis in A549 Cells

**Figure 3:**
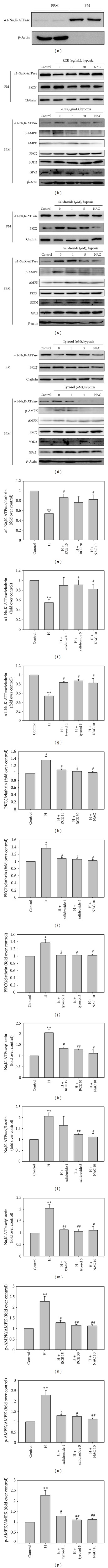
RCE, salidroside, and tyrosol prevent hypoxia-mediated Na,K-ATPase endocytosis in A549 cells. The analysis of the distribution of *α*1-Na,K-ATPase and *β*-actin in the plasma membrane (PM) and post-plasma membrane (PPM) fractions by western blotting (a). The A549 cells were exposed to hypoxia (1% O_2_) for 24 h, and the expressions of *α*1-Na,K-ATPase, PKC*ζ*, p-AMPK, SOD2, and GPx2 were analyzed with or without a 30 min pretreatment with RCE (b), salidroside (c), and tyrosol (d) in the PM and PPM. Clathrin served as a loading control. (e)–(g) and (h)–(j) are the quantitative data for the relative levels of *α*1-Na,K-ATPase and PKC*ζ* in the PM from (b), (c), and (d). (k)–(m) and (n)–(p) are the quantitative data for the relative levels of *α*1-Na,K-ATPase and p-AMPK in the PPM from (b), (c), and (d). The results represent the mean ± SEM of three independent experiments. *P < 0.05; **P < 0.01; ***P < 0.001 versus control; ^#^ *P* < 0.05; ^##^ *P* < 0.01; ^###^ *P* < 0.001 versus hypoxia (H).

 To identify whether the method we used is applicable to separate the PM and PPM fractions, *α*1-Na,K-ATPase and *β*-actin were used as the specific protein markers for the PM and PPM fractions, respectively. The results shown in Figure 3(a) indicate that the two fractions were clearly separated, as there is no *β*-actin in the PM and the *α*1-Na,K-ATPase is predominantly in the PM fraction. Exposure to hypoxia significantly decreased the abundance of the *α*1-Na,K-ATPase in the PM in A549 cells (0.54 ± 0.04-fold compared to control; *P* < 0.01, in Figures 3(b), 3(c), and 3(d)); pretreatment with RCE at different doses (15 and 30 *μ*g/mL) effectively blunted the *α*1-Na,K-ATPase endocytosis in response to hypoxia (0.85 ± 0.09 and 0.70 ± 0.17-fold compared to the control, in Figure 3(b)) with a significant difference at the dose of 15 *μ*g/mL (*P* < 0.05). Salidroside (1.0 and 5.0 *μ*M) also showed similar efficacy for the reduction of *α*1-Na,K-ATPase in the PM under hypoxia (0.90 ± 0.16 and 0.91 ± 0.10-fold compared to the control) with optimal efficacy at a dose of 5.0 *μ*M (*P* < 0.05). Similarly, tyrosol, which is the aglycone of salidroside, exerted potent protective effects comparable to salidroside (0.83 ± 0.02 and 0.87 ± 0.03-fold compared to control), especially at doses of 1.0 and 5.0 *μ*M (*P* < 0.05). NAC (10 *μ*M) was used as the positive control and displayed a potent inhibitory effect on the hypoxia-induced *α*1-Na,K-ATPase endocytosis as expected (0.83 ± 0.10-fold compared to control, *P* < 0.05). To investigate the underlying mechanisms of the effect of RCE, salidroside, and tyrosol on hypoxia-induced Na,K-ATPase endocytosis in A549 cells, we assessed the expression of PKC*ζ* in the PM in response to hypoxia. As shown in Figures 3(b), 3(c), and 3(d), the hypoxia-mediated decrease in *α*1-Na,K-ATPase is accompanied by the increased translocation of PKC*ζ* to the PM (1.37 ± 0.09-fold compared to control; *P* < 0.05). Meanwhile, we also observed that hypoxia significantly increased the phosphorylation of AMPK at T172 in the PPM fraction (2.29 ± 0.23-fold compared to control; *P* < 0.01). Furthermore, the protein abundance of *α*1-Na,K-ATPase was found to be increased in the PPM fractions under hypoxia (2.06 ± 0.16-fold compared to control; *P* < 0.01, in Figures 3(b), 3(c), and 3(d)). However, preincubation with RCE (15 and 30 *μ*g/mL) ameliorated both the *α*1-Na,K-ATPase endocytosis and the translocation of PKC*ζ* to the PM (1.09 ± 0.03 and 1.05 ± 0.01-fold compared to control, resp., *P* < 0.05) in addition to attenuating the increased level of phosphorylated AMPK (p-AMPK) in response to hypoxia (1.29 ± 0.10 and 1.16 ± 0.03-fold compared to control, resp., *P* < 0.05 and *P* < 0.01; Figure 3(b)). Moreover, salidroside and tyrosol (1.0 and 5.0 *μ*M) also showed similar efficacies in alleviating increased p-AMPK (1.30 ± 0.05 and 1.25 ± 0.04-fold compared to control, resp., *P* < 0.05; 1.30 ± 0.10 and 1.11 ± 0.07-fold compared to control, resp., *P* < 0.05 and *P* < 0.01) and PKC*ζ* translocation (1.08 ± 0.03 and 1.06 ± 0.02-fold compared to control, resp., *P* < 0.05; 1.03 ± 0.04 and 1.03 ± 0.01-fold compared to control, resp., *P* < 0.05) under hypoxia (Figures 3(b) and 3(c)). Consistently, NAC also showed significant efficacy in attenuating translocated PKC*ζ* (1.02 ± 0.03-fold compared to control; *P* < 0.05) and increased p-AMPK (1.13 ± 0.06-fold compared to control; *P* < 0.01) in response to hypoxia. On the other hand, there was no significant inductive effect on major cellular antioxidant enzymes, including SOD2 and GPx2, under hypoxic conditions, despite the fact that these enzymes were reported to provide beneficial effects for Na,K-ATPase against hypoxic insults [8].

### 3.3. The Protective Effects of RCE, Salidroside, and Tyrosol Are Associated with Alleviating AMPK-PKC*ζ* Pathway under Hypoxia

**Figure 4:**
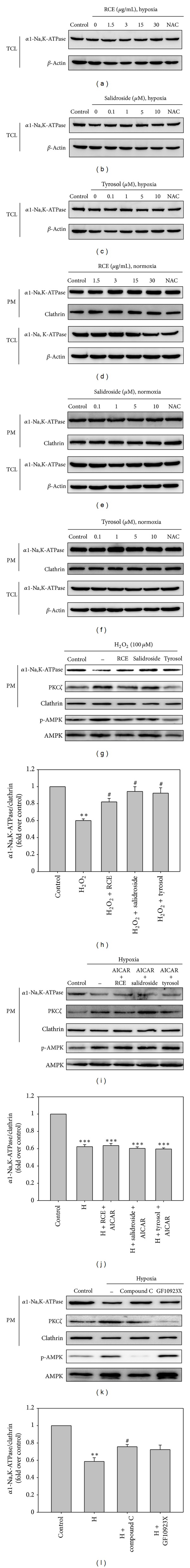
The protective effects of RCE, salidroside, and tyrosol are associated with alleviating the AMPK-PKC*ζ* pathway under hypoxia. The cells were pretreated for 30 min with the indicated concentrations of *Rhodiola* products or chemicals under hypoxia (1% O_2_) or normoxia for 24 h. The expressions of *α*1-Na,K-ATPase were analyzed in the presence or absence of RCE (a), salidroside (b), and tyrosol (c) in total cell lysate (TCL) under hypoxia or RCE (d), salidroside (e), and tyrosol (f) in both PM and TCL under normoxia. Cells were pretreated with RCE (15 *μ*g/mL), salidroside (5.0 *μ*M), or tyrosol (5.0 *μ*M) for 30 min and then exposed to H_2_O_2_ (100 *μ*M) for another 30 min under normoxia (g). AICAR (2 mM) was added in combination with RCE (15 *μ*g/mL), salidroside (5.0 *μ*M), or tyrosol (5.0 *μ*M) (i). Compound C (2 *μ*M) or GF10923X (1 *μ*M) was preincubated alone prior to hypoxic exposure (k). (h), (j), and (l) are the quantitative data for the relative levels of *α*1-Na,K-ATPase in the PM from (g), (i), and (k). The results represent the mean ± SEM of three independent experiments. *P < 0.05; **P < 0.01; ***P < 0.001 versus control; ^#^ *P* < 0.05; ^##^ *P* < 0.01; ^###^ *P* < 0.001 versus hypoxia (H) or H_2_O_2_.

We observed that hypoxia altered the expression of *α*1-Na,K-ATPase in both PM and PPM fractions (Figures 3(b), 3(c), and 3(d)); however, the total amount of *α*1-Na,K-ATPase in the cell lysate remained unchanged in response to hypoxia (Figures 4(a), 4(b), and 4(c)). The results confirmed that hypoxia indeed triggered *α*1-Na,K-ATPase endocytosis from the PM to intracellular compartments. We also showed that the effects of *Rhodiola *products are specific for hypoxia-induced Na,K-ATPase endocytosis, because they show no significant changes on *α*1-Na,K-ATPase expression in both PM and the total cell lysate (TCL) under normoxia (Figures 4(d), 4(e), and 4(f)). To further clarify the signal mechanisms of RCE and its bioactive compounds for the preservation of *α*1-Na,K-ATPase through the ROS-AMPK- PKC*ζ* pathway, the effect of hydrogen peroxide (H_2_O_2_) was investigated under normoxia. We found that H_2_O_2_ (100 *μ*M) significantly decreased the abundance of *α*1-Na,K-ATPase in PM in normoxia (0.60 ± 0.02-fold compared to control; *P* < 0.01). However, RCE, salidroside, and tyrosol effectively prevented the Na,K-ATPase endocytosis (0.82 ± 0.04, 0.94 ± 0.06, and 0.92 ± 0.06-fold compared to control, resp., *P* < 0.05) response to application of H_2_O_2_ in normoxia (Figure 4(g)). Furthermore, the effect of compound C (an AMPK inhibitor), AICAR (5-amino-1-*β*-D-ribofuranosyl-imidazole-4-carboxamide, an AMPK activator), and GF10923X (a PKC inhibitor) were also examined. Pre-incubation with AICAR abolished the protective effect of RCE, salidroside, and tyrosol on the hypoxia-mediated *α*1-Na,K-ATPase endocytosis (0.64 ± 0.02, 0.60 ± 0.02 and 0.59 ± 0.01-fold compared to control, resp.; *P* < 0.001), which was accompanied by increased p-AMPK and PKC*ζ* translocation in the PM fraction (Figure 4(i)). In addition, pretreatment of the cells with compound C or GF10923X effectively attenuated the *α*1-Na,K-ATPase endocytosis (0.76 ± 0.03 and 0.72 ± 0.05-fold compared to control, resp.; *P* < 0.05 and *P* = 0.11) via the suppression of p-AMPK or translocated PKC*ζ*, respectively (Figure 4(k)). These results indicate that increased p-AMPK and translocated PKC*ζ* are required for the endocytosis of Na,K-ATPase under hypoxic conditions in A549 cells. 

### 3.4. RCE Confers a Similar Preventive Effect and Mechanism on Hypoxia-Mediated Reduction of Na,K-ATPase in a Hypobaric Hypoxia Rat Model

**Figure 5:**
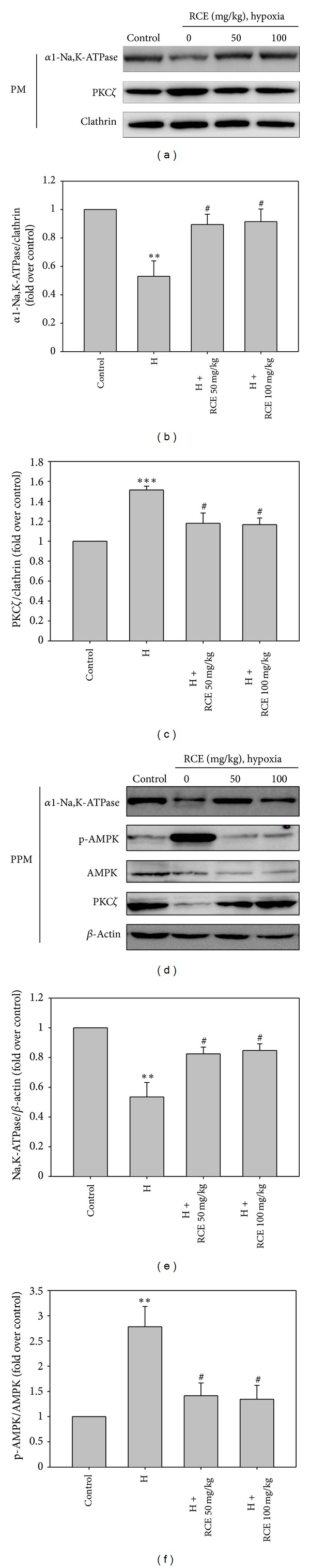
RCE confers a similar preventive effect on the hypoxia-mediated reduction of Na,K-ATPase via a similar mechanism in a hypobaric rodent model. The rats were exposed to simulated hypobaric hypoxia (8000 m) for 9 h in the presence or absence of RCE. The expression of *α*1-Na,K-ATPase and PKC*ζ* in the PM isolated from the right upper lobe of the lung (a) and the relative levels of *α*1-Na,K-ATPase (b) and PKC*ζ* (c) were quantified. The expression of *α*1-Na,K-ATPase and p-AMPK in the PPM (d) and the relative levels of *α*1-Na,K-ATPase (e) and p-AMPK (f) were quantified. The results represent the mean ± SEM of four independent experiments. *P < 0.05; **P < 0.01; ***P < 0.001 versus control; ^#^ *P* < 0.05; ^#^ *P* < 0.01; ^###^ *P* < 0.001 versus hypoxia (H).

To verify the effect of RCE *in vivo*, a hypobaric hypoxia rat model was employed. The results indicate a significantly decreased protein abundance of *α*1-Na,K-ATPase in both the PM (0.53 ± 0.11-fold over control with *P* < 0.01, Figures 5(a) and 5(b)) and PPM (0.54 ± 0.10-fold over control with *P* < 0.01, Figures 5(d) and 5(e)) fractions of rat lung tissue lysates in response to hypoxia. Hypoxia also significantly increased the level of PKC*ζ* in the PM (1.51 ± 0.04-fold compared to control with *P* < 0.001, Figures 5(a) and 5(c)), which was accompanied by increased p-AMPK (2.78 ± 0.40-fold compared to control with *P* < 0.01, Figures 5(d) and 5(f)) similar to the results observed in A549 cells. Nevertheless, pretreatment with RCE (50 or 100 *μ*g/kg) effectively alleviated both the PKC*ζ* translocation in the PM (1.18 ± 0.10 and 1.17 ± 0.07-fold compared to control, resp., *P* < 0.05) and the increased p-AMPK levels (1.42 ± 0.25 and 1.35 ± 0.27-fold over control, resp., *P* < 0.05). RCE also preserved the abundance of *α*1-Na,K-ATPase in both the PM (0.90 ± 0.07 and 0.92 ± 0.09-fold over control, resp., *P* < 0.05) and PPM (0.83 ± 0.04 and 0.85 ± 0.05-fold over control, resp., *P* < 0.05) during hypoxia. 

## 4. Discussion

Salidroside and tyrosol are regarded as the major bioactive compounds and are used as the standard markers for the quantitative analysis of the quality of different *Rhodiola *species or preparations [24]. Until now, most studies have focused on the role of salidroside, rather than tyrosol, in the efficacy of *Rhodiola* species. Thus, intensive studies about tyrosol are still lacking. In this study, we found that tyrosol, an antioxidant phenolic compound, showed antioxidant properties comparable to those of salidroside in the regulation of Na,K-ATPase via similar protective mechanisms. These results suggest that tyrosol may play a significant role in the protective effects of* Rhodiola crenulata *against oxidative-stress-associated disorders. It should be noted that tyrosol also exists in wine and olive oils, two representative components of the Mediterranean diet, which have been reported to have antioxidant, anticancer, and cardiovascular benefits [25, 26]. Thus, it is interesting to investigate whether salidroside and tyrosol have any beneficial effect for the prevention of cancer or cardiovascular disease. 

The present study shows for the first time that salidroside and tyrosol exert preventive effects on *α*1-Na,K-ATPase endocytosis in A549 cells during hypoxia with efficacies comparable to RCE. We also clarified the underlying mechanisms of RCE and its bioactive components by showing that they are associated with counteracting the ROS-AMPK-PKC*ζ* pathway. Using a hypobaric hypoxia rodent model, we further found that hypoxia-induced molecular responses similar to those shown in A549 cells and RCE exerted similar protective effects via similar mechanisms. A549, which displays many characteristics of alveolar epithelial cells, is a generally accepted model of ion transport which displays great similarity to the primary rat alveolar type II (ATII) cells in response to hypoxia [27, 28]. The trafficking of Na,K-ATPase under hypoxia is endocytosis from the plasma membrane to intracellular compartments first and then degradation [1]. Our results showed more rapid Na,K-ATPase degradation under hypoxia in rat lungs than in A549 cells (Figures 5(a) and 5(d)), which is consistent with previous studies [8, 29]. The results suggest that different rates of Na-K-ATPase degradation in response to hypoxia may exist between A549 cells and rat ATII cells. Even so, Na,K-ATPase endocytosis in both A549 and rat ATII cells is primarily through ROS-AMPK-PKC*ζ* pathways [14, 30]. Thus, the results of A549 cells should be generally transferable to the* in vivo* situation. 

Previous evidence indicated that diminishing mitochondrial ROS via antioxidants or mitochondrial inhibitors prevented hypoxia-induced decreases in both the expression and activity of Na,K-ATPase [13]. In the present study, we show that RCE and its bioactive components significantly alleviated both the production of intracellular ROS and Na,K-ATPase endocytosis in response to hypoxia. Furthermore, we also ruled out the possibility that the reduction of intracellular ROS burden was due to cytotoxicity imparted by RCE, salidroside, and tyrosol. In addition, we also showed that *Rhodiola* products all significantly prevented H_2_O_2_-induced *α*1-Na,K-ATPase endocytosis (Figure 4(g)). The anti-ROS effect of these *Rhodiola* products is consistent with previous studies that found that salidroside is able to decrease the oxidative stress induced by CoCl_2_, which is a hypoxia-mimicking agent, and showed protective effects against H_2_O_2_ insults in different cell lines [31, 32]. Combined with the previous findings, our results demonstrate that *Rhodiola* products exhibit potency in alleviating oxidative stress in different hypoxic models. The antioxidant NAC, which is used as a positive control in this study, showed antioxidant activity to alleviate the activation of the AMPK-PKC*ζ* pathway and *α*1-Na,K-ATPase endocytosis under hypoxic conditions. Furthermore, NAC was also reported to attenuate oxidative stress and preserved Na,K-ATPase activity in an oleic-acid-induced lung injury rodent model due to its antioxidant properties [33]. Thus, our findings suggest that the protective effect of these *Rhodiola* products is associated with their antioxidant activity. These results also imply that supplementation with antioxidants might help people working at high altitudes maintain AFC.

AMPK is a critical metabolic sensor for energy homeostasis that regulates a large number of downstream targets. AMPK has also been shown to play a key role in the regulation of Na,K-ATPase endocytosis via the phosphorylation and recruitment of PKC*ζ* during hypoxia [14]. To identify the role of AMPK in the protective effects of *Rhodiola *products, the effects of AICAR and compound C were examined in the present study. Our results showed that compound C completely inhibited p-AMPK and alleviated the *α*1-Na,K-ATPase endocytosis in response to hypoxia (Figure 4(k)). In contrast, AICAR abolished the protective effect of RCE, salidroside, and tyrosol, which was accompanied by increased p-AMPK levels and PKC*ζ* translocation. In addition to the AMPK signal modulators, pretreatment with GF10923X also inhibited the translocation of PKC*ζ* to PM and ameliorated hypoxia-mediated *α*1-Na,K-ATPase endocytosis (Figure 4(i)). It should be noted that GF10923X did not inhibit the level of p-AMPK, confirming that the translocation of PKC*ζ* is a downstream consequence of AMPK phosphorylation under hypoxia. The findings suggest that *Rhodiola* products prevented the hypoxia-induced Na,K-ATPase endocytosis mediated by the AMPK-PKC*ζ* pathway. However, the effects of *Rhodiola* products for AMPK are controversial. *Rhodiola rosea *and salidroside were shown to activate the AMPK pathway to regulate the cellular energy status in different cell lines [34, 35]. This implied that *Rhodiola *products may trigger energy production by activating AMPK when organisms are in low-energy stress. In contrast, the present study shows that RCE and its bioactive components all attenuated hypoxic stress and intracellular ROS burden and, in turn, decreased the overexpressed p-AMPK. It is known that different pathways exist for AMPK activation, and the hypoxia-induced activation of AMPK is thought to be primarily driven by intracellular ROS rather than energy status [36]. The contrary effects of *Rhodiola* products for AMPK activation in different experimental conditions might be due to its adaptogen property, which normalizes body functions, allows the organism to adapt to environmental stresses, and prevents stressful insults from such factors [15, 37, 38]. Thus, *Rhodiola* species,which are phytoadaptogens, might act as bifunctional modulators for certain signal targets, such as AMPK, in response to different cellular conditions. 

**Figure 6:**
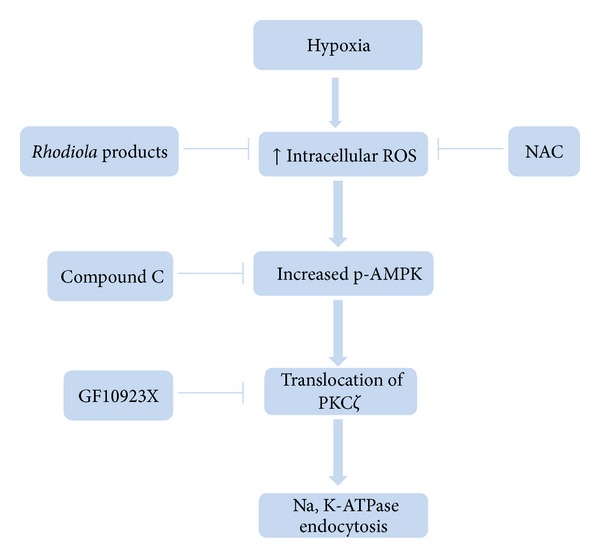
The proposed mechanism of *Rhodiola crenulata* in attenuating the hypoxia-mediated Na,K-ATPase endocytosis. RCE and its bioactive components attenuate the hypoxia-induced *α*1-Na,K-ATPase endocytosis by inhibiting the ROS-AMPK-PKC*ζ* pathway.

 Although RCE and its bioactive components show excellent antioxidant potency, the expression level of the primary antioxidant enzymes, such as SOD2 and GPx2, was not changed in our experiment (Figure 3). These results are consistent with the previous study, which indicated that *Rhodiola rosea *can directly ameliorate the H_2_O_2_-induced oxidative stress but not through the activation of SOD and catalase [39]. Thus, our results imply that the antioxidant activity of *Rhodiola *species does not primarily result from the induction of antioxidant enzymes. These findings also agree with the great radical scavenging activity of RCE, salidroside, and tyrosol, as indicated by the elimination of the 2,2-diphenyl-1-picrylhydrazyl (DPPH) radical, which is a stable radical that is widely used to test the efficacy of compounds as free-radical scavengers [19]. Thus, we suggest that RCE and its bioactive components attenuate the hypoxia-mediated oxidative stress via their function as ROS scavengers instead of through the induction of antioxidant enzymes. Combining the evidence from our cell and rodent models, we propose that the preventive effect of RCE, salidroside, and tyrosol for hypoxia-mediated Na,K-ATPase endocytosis is mediated through the alleviation of the generation of intracellular ROS, which in turn, decreases the p-AMPK level and PKC*ζ* translocation to the PM (Figure 6). 

Alveolar hypoxia is not only a pathophysiological process in HAPE but also in other inflammatory-associated respiratory disorders, such as acute lung injury and acute respiratory distress syndrome, and the severity of these disorders is related to the dysfunction of Na,K-ATPase [9–11]. Similarly, hyperoxia-induced oxidative stress was also shown to inhibit Na,K-ATPase activity and AFC [7]. In the present study, *Rhodiola *products showed remarkable efficacy on *α*1-Na,K-ATPase against hypoxic insults. Combined with anti-inflammatory activity [37], whether it has any beneficial effects on other hypoxia-associated respiratory disorders requires further elucidation, as there are many other ion channels that are known to be involved in transalveolar fluid transport, including ENaC [1, 4]. Thus, it would be interesting to investigate whether RCE is effective with other ion channels or against hyperoxia-impaired Na,K-ATPase function. It should be noted that the present study was carried out with some limitations, such as the fact that the measurement of ROS production as hypoxia-induced ROS production diminished after reoxygenation for 30 minutes [13]. Thus, the measurement needs to be processed as soon as possible in order to avoid disturbance due to reoxygenation. In addition, we showed that *Rhodiola *products and NAC almost completely alleviated the hypoxia-induced ROS production (Figures 2(a), 2(b), and 2(c)), but they restored only about 80–90% of Na,K-ATPase expression (Figures 3(b), 3(c), and 3(d)). Combined with the results of compound C and GF10923X (Figure 4(d)), the findings indicated that blocking ROS-AMPK-PKC*ζ* pathway is not able to blunt Na,K-ATPase endocytosis completely. Our results suggest that ROS-AMPK-PKC*ζ* is the major, but not the only, signal pathway involved in the hypoxia-reduced Na,K-ATPase endocytosis. However, it still needs to be further elucidated.

In conclusion, the present study provides significant evidence to support the ethnopharmacological role of* Rhodiola crenulata *as a popular folk medicine for high-altitude sickness in Tibet, and this effect is at least partly due to the ability to maintain pulmonary sodium transport via the preservation of Na,K-ATPase.
